# New evidence of the emergence of the East Asian monsoon in the early Palaeogene

**DOI:** 10.1038/s41598-022-24298-z

**Published:** 2022-11-28

**Authors:** Li-Fei Su, Qian-Qian Zhang, Yan-Kun Sun, Shao-Liang Zhang, Thierry Smith, Cheng-Sen Li

**Affiliations:** 1 College of Resource and Environment, Northeast Agricultural University, Harbin, 150030 Heilongjiang China; 2 Directorate Earth and History of Life, Royal Belgian Institute of Natural Sciences, Brussels, Belgium; 3 State Key Laboratory of Systematic and Evolutionary Botany, Institute of Botany, Chinese Academy of Sciences, Xiangshan, Beijing People’s Republic of China

**Keywords:** Palaeontology, Palaeoclimate

## Abstract

Previous palaeoenvironmental reconstructions have implied that East Asia was dominated by a zonal climate pattern during the Eocene, with an almost latitudinal arid/semiarid band at ~ 30° N. However, this long-standing model has recently been challenged by growing body of multidisciplinary evidence. Some studies indicated that central China was characterized by climatic fluctuations between humid and drier conditions during the Early Eocene, akin to the present East Asian monsoon (EAM) regime. Using palynological assemblages in the Tantou Basin, central China, we quantitatively reconstructed climate changes from the Late Palaeocene to Early Eocene to better understand climate change in central China. Palynological assemblages revealed that the coniferous and broad-leaved mixed forest in this area received no less than 800 mm of annual precipitation and experienced a climate change from warm and wet to relatively cool and dry. According to palaeoclimate curves, a sudden climate change occurred in the Early Eocene, with the mean annual temperature and precipitation decreasing by 5.1 °C and 214.8 mm, respectively, and the climate became very similar to the present climate, which is controlled by the monsoon. Therefore, this significant climate change during the Early Eocene may signal the emergence of the EAM in East Asia.

## Introduction

Over the past decades, the origin of the East Asian monsoon has long been of great scientific interest. Numerous palaeoclimate records from fossils, sediment analysis and model simulations have contributed to our understanding of the monsoon climate system. One of the outstanding issues in monsoon studies is the age of the East Asian monsoon system^1^. However, due to the limitations of palaeoenvironmental records, the origin of the East Asian monsoon is still the subject of intense debate in the scientific community^2^. There are three main opinions regarding the first emergence of the modern East Asian monsoon: the first opinion is that the East Asian monsoon climate may have existed in the Early Eocene^3,4^; the second one is that it may have existed in the Middle or Late Eocene^5–7^; and the last one is that it may have existed in the Oligocene^1^.

However, the debate on the Early Eocene climate dominated whether by zonal climate or non-zonal monsoon climate patterns has persisted for decades. Evidence from stable isotope analysis indicates that the Early Eocene (~ 55 Ma) atmospheric circulation in Asia was already very similar to that of today^4^. Based on multidisciplinary evidence, Quan et al.^2^ analyzed the Early Eocene climate of central and eastern China and indicated that it would have been much drier if it had been controlled by a planetary wind system. Their results suggested that a monsoonal or monsoon-like climate might have prevailed over China during the Early Eocene. Li et al.^8^ studied two models (the low-resolution Norwegian Earth System Model and the high-resolution Community Atmosphere Model version 4) to simulate the Early Eocene climate of China and supported the presence of a zonal/zonal-like arid desert/steppe climate band. In addition, Based on plant fossils, Xie et al.^9^ analyzed the Early Eocene climate of southern China and indicated that an extremely arid climate (mean annual precipitation < 230 mm) dominated the region. In general, to further determine the climate pattern of the Early Eocene, substantial evidence is still needed.

Understanding the initiation of the current monsoon climate system is vital not only for enriching the knowledge of monsoon history but also for recognizing the mechanism of its variations^1^. Palaeophytogeography is considered a very reliable method for studying Cenozoic global climate evolution. Numerous authors have used palaeobotanical methods to study climatic conditions during the Cenozoic, especially the early Palaeogene. Although a large number of previous studies have quantitatively reconstructed the palaeoclimate during the early Palaeogene, especially the Late Palaeocene to Early Eocene, and have identified significant global warming events during this period, only a few of them have explored the relationship between the palaeoclimate during this period and the monsoon climate^10–15^. In addition, data are still lacking on the terrestrial ecosystem response to climate changes during the Late Palaeocene to Early Eocene and its effects on global climate patterns during the early Palaeogene. Petroleum explorations of non-marine deposits in the Tantou Basin, Luanchuan (LC) County, Henan Province, central China, have contributed to the accumulation of an enormous amount of palynological data, which are potential sources of palaeoclimate evidence for monsoon climate studies.

Palaeobotany research in LC region began in 1984. Wang et al.^16^ studied the palynological flora and divided the lower Tertiary sediments of the Tantou Basin into three palynological assemblages. Wang et al.^17^ reported three new genera and eleven new species of angiosperms from the Palaeocene–Eocene strata in the Tantou Basin in LC. Mao et al.^18^ concluded that the palaeoclimate changed from a semihumid subtropical to a semihumid warm temperate climate, which indicated that the palaeoclimate became wetter during the Palaeogene. Most importantly, although the palynological assemblages in LC region during the Late Palaeocene–Early Eocene have been reported before, the discussion of palaeoclimate is still in the stage of qualitative description. Therefore, quantitative research on changes in the climate and environment during the Late Palaeocene to Early Eocene in LC is urgently needed. Our primary objective in this study is to provide a reliable baseline for studies on global vegetation succession and climate changes during this time. By applying the coexistence approach (CA) based on palynological assemblages, we reconstructed the curves of palaeoclimate changes throughout the Late Palaeocene to Early Eocene. Our results indicated that the palaeoclimate during that time was warmer and wetter than the present climate, which is consistent with the findings of other studies^19,20^. In addition, the quantitative reconstruction of the palaeoclimate in the Tantou Basin in the LC region, central China, during the Late Palaeocene to Early Eocene revealed that climate fluctuations during this period showed obvious monsoon climate characteristics.

## Geographic and geological setting

### Geographic setting

**Figure 1:**
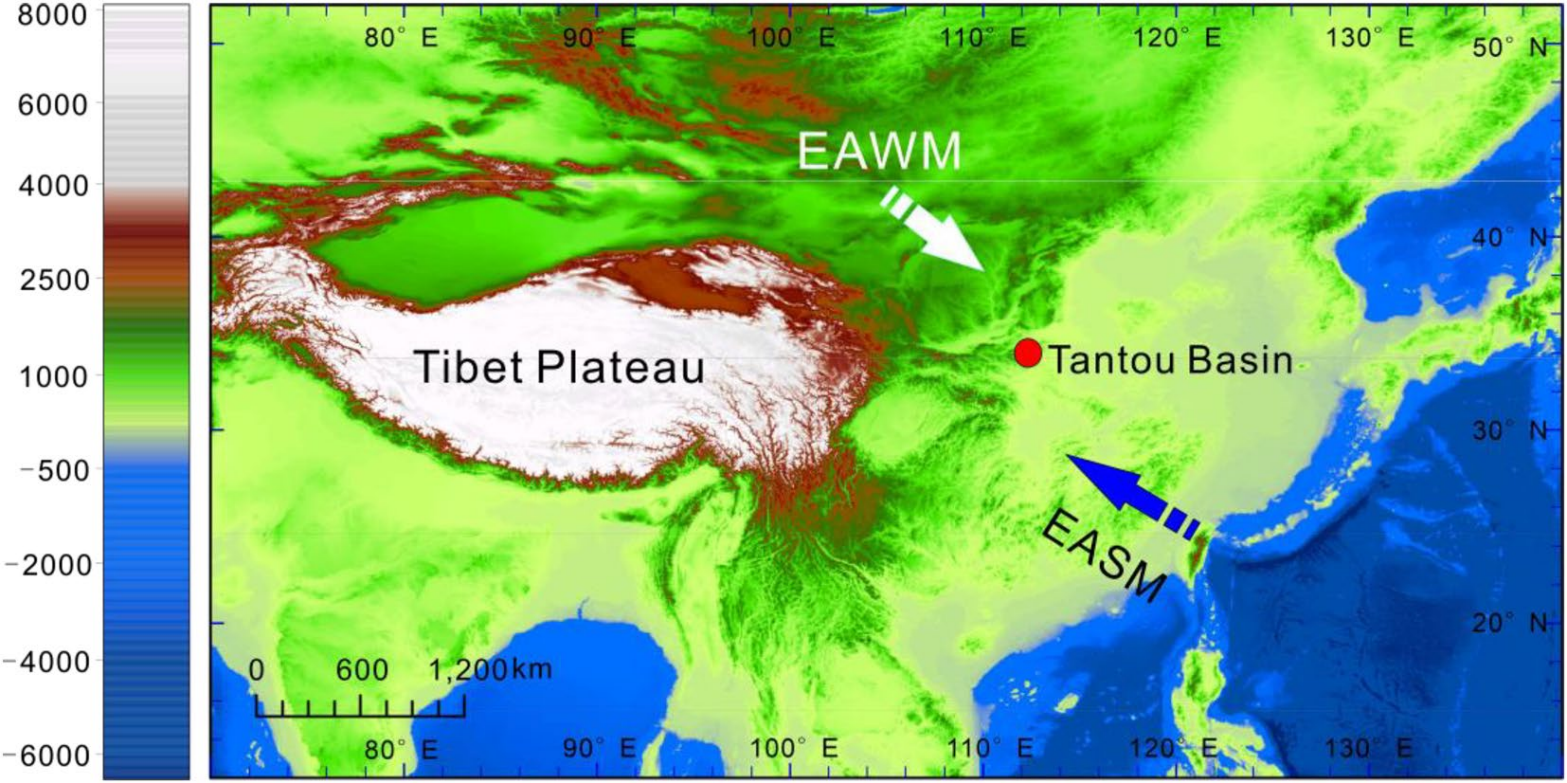
Map showing the study site in the Tantou Basin. ArcMap 10.2 software (http://www.tuicool.com/articles/VfaMfy) was used to create this figure.

The LC Sect. (33° 39ʹ–34° 11ʹ N, 111° 11ʹ –112° 01ʹ E) is situated in southern Luoyang city, western Henan Province, central China (Fig. 1). LC is currently dominated by a warm temperate continental monsoon climate^21^. The mean annual temperature (MAT) is 12.2 °C, the mean temperature of the coldest month (CMMT) is 8 °C, and the mean temperature of the warmest month (WMMT) is 24.2 °C. The mean annual precipitation (MAP) is 880 mm, the maximum monthly precipitation (MMaP) is 205.5 mm, and the mean minimum monthly precipitation (MMiP) is 9.9 mm^22^. The LC area features mountainous and hilly terrain, and the local typical vegetation is mountainous deciduous forest and warm coniferous forest. Common coniferous trees in the flora of this region include *Pinus tabuliformis*, *Pinus bungeana* and *Pinus armandii.* Common broad-leaved elements include *Betula platyphylla*, *Populus tomentosa*, *Zelkova serrata, Corylus,* and *Ulmus*. Subtropical plants, such as *Cercidiphyllum japonicum*, *Betula albosinensis* and *Lindera obtusiloba*, also have a certain distribution in this region^21,23^.

### Geological setting

The Tantou Basin in central China contains exposures of continuous Mesozoic-Cenozoic deposits and has been the subject of detailed stratigraphic surveys^16,24–26^. The sediments in the Tantou Basin are divided into four formations from bottom to top: the Qiupa Formation (Fm.), the Gaoyugou Fm., the Dazhang Fm., and the Tantou Fm., the divisions of which are supported by palaeontology^16,24–27^, petrology^28^, and sedimentology^29,30^.

The Qiupa Fm. is assigned to the Late Cretaceous based on the discovery of a large number of dinosaur egg fossils^26,27^. Based on the discovery of standard Middle Palaeocene mammal fossils, such as Bemalambda and Mesonychidae, and comparison with other Middle Palaeocene lithologies, the Gaoyugou Fm. is generally considered to be a Middle Palaeocene stratum^24,25,31,32^. Abundant fossils, such as *Physa* spp., *Melania* sp., *Eupera* sp., *Cyprinotus speciosus Mandel*, *Metacyri* ssp., *Eucyris* sp., *Cyclocypris* sp., and *Cypris decaryi Gautheir*, have been found in the Dazhang Fm^26^. Some fossils, such as *Parhydrobia xiaohegouensis* Li and *Opeas guangdongensis* Yu, are standard Palaeocene fossils, while mammalian fossils, such as Pastoraledontidae and Pseudictopidae, are standard Late Palaeocene mammal fossils, so the Dazhang Fm. is considered to be Late Palaeocene in age^24–26^. In addition, the sediment age of the Dazhang Fm. is also supported by palynology based on the appearance of a large number of Palaeocene/Eocene palynomorphs, such as Ulmaceae and Proteaceae, which further support a Late Palaeocene age for the Dazhang Fm.. Numerous faunal fossils have been found in the Tantou Fm., including Prodinoceratinae and Archaeolambdidae, indicating that the sediments were deposited in the Early Eocene^24,26^. Furthermore, the sediment age of the Tantou Fm. is also supported by palynology based on the appearance of Early Eocene palynomorphs, such as *Tricolpopollenites* and *Monocolpopollenites*^16^. The precise age of the Tantou Basin remains ambiguous due to a lack of paleomagnetic or U‒Pb dating of the sedimentary succession. However, based on a comprehensive comparison of stratigraphic and lithological characteristics, the sediments of the Gaoyugou Fm., Dazhang Fm. and Tantou Fm. exposed in the section were most likely deposited during the Middle Palaeocene, Late Palaeocene and Early Eocene, respectively.

**Figure 2:**
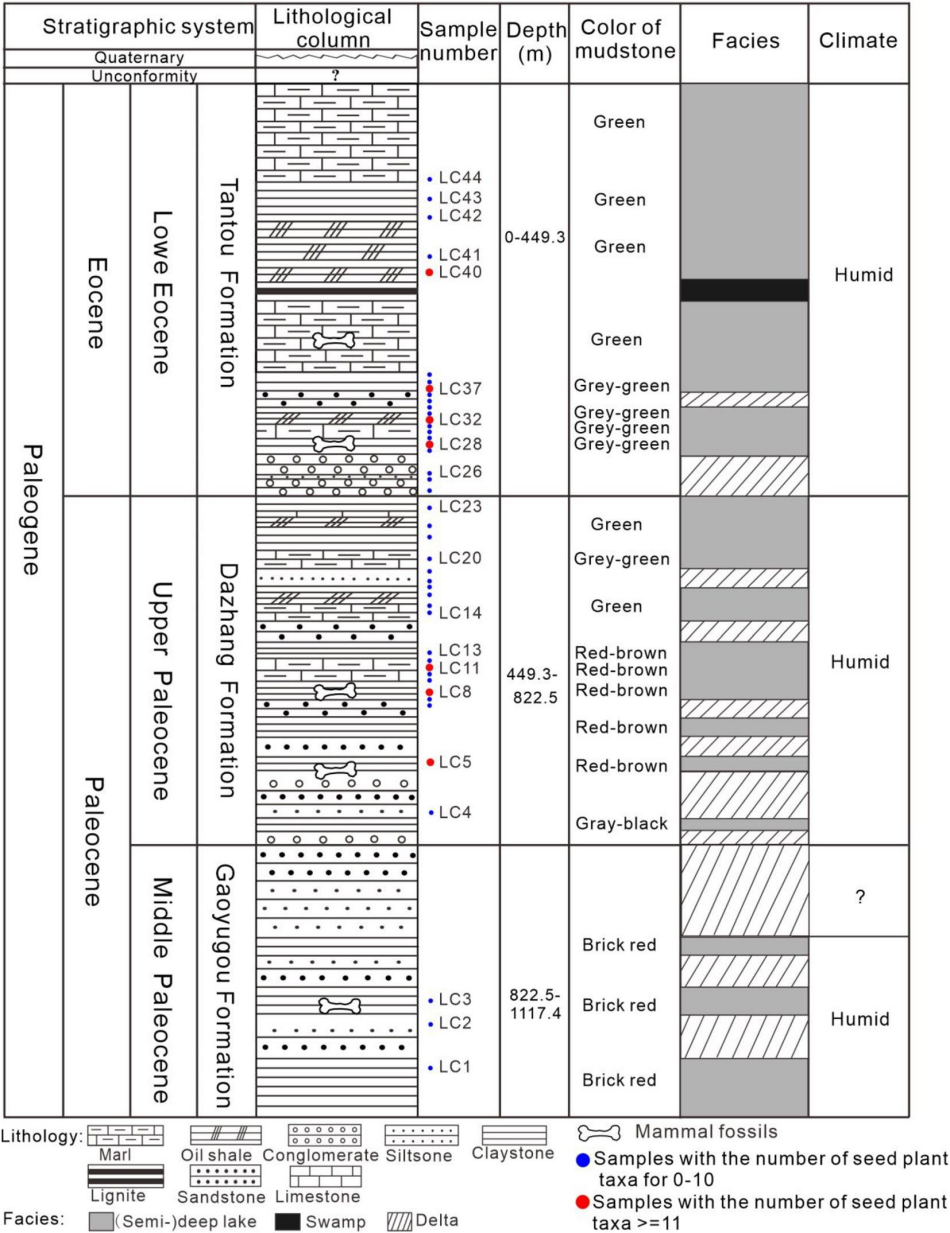
Sedimentary succession, geomorphic and lithologic characteristics, and sampling locations of the Luanchuan section in the Tantou Basin (modified from Du and Chen^24^; Tong and Wang^25^ and the climate mainly refers to Wang et al.^33^).

The lithology of the Gaoyugou Fm. is mainly composed of sandstone and brick-red claystone, reflecting the strong oxidizing environment in its diagenetic history^18,33^. The main lithology of the Dazhang Fm. is marl, oil shale, claystone and glutenite interbedded. The lithology of the Tantou Fm. is mainly composed of marl, claystone, oil shale with a small amount of siltstone and conglomerate. No evaporative salt debris is found in Dazhang and Tantou Fm.s. These sedimentary features of them reflect relatively mild and humid climatic conditions^33^. The detailed lithological features, thicknesses and climatic characteristics from lithological reactions of each formation are shown in Fig. 2.

## Results

### Palynological assemblages

No palynological samples were found in the LC1–3 samples. Palynomorphs were found only in the Dazhang and Tantou Formations.

**Figure 3:**
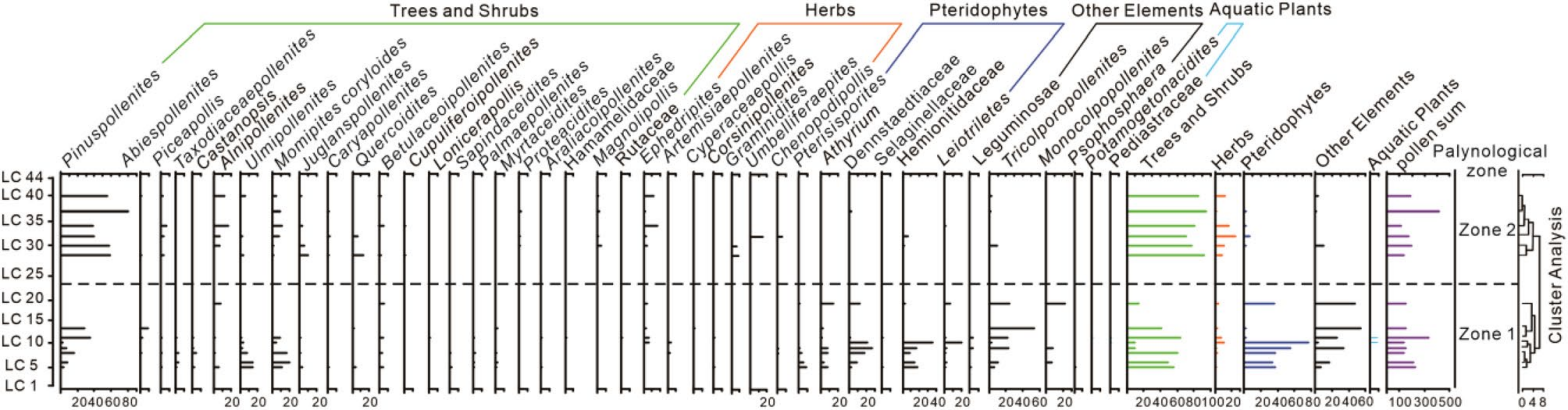
The percentages of pollen in the Luanchuan section.

We identified 48 palynomorph species and observed 2717 palynomorph grains across the whole section; these palynomorphs consisted of 27 trees and shrubs (61.76%), 8 herbs (6.74%), 6 pteridophytes (17.60%), 5 ambiguous elements (13.69%) and 2 aquatic plants (0.14%) (Table S1). Based on the ecological requirements of each palynomorph, 13 thermophilic elements, 3 cold-tolerant elements, 3 xerophilous elements and 5 hygrophilous elements were observed (Table 1). Of the 44 collected samples, 14 contained enough palynomorphs to perform the TILIA analysis by recognizing two zones from bottom to top based on the RA (Fig. 3).

**Table 1:** List of the Luanchuan taxa grouped by ecological requirements and RA in the whole section and in the two palynological zones from the Late Palaeocene to Early Eocene (table style refers to Jiménez-Moreno^34^).

Palynomorph	The whole section	Zone 1 (%)	Zone 2 (%)
**Thermophilic elements**	6.03	7.61	2.78
*Taxodiaceaepollenites*	0.44	0.8	/
*Palmaepollenites*	2.54	4.61	/
*Sapindaceidites*	0.37	0.67	/
*Myrtaceidites*	0.33	0.6	/
*Hamamelidacidites*	0.29	0.53	/
*Araliaceoipollenites*	0.07	0.13	/
*Huodendron*	0.07	0.13	/
*Rutaceoipollis*	0.04	0.07	/
*Castanopsis*	0.7	0.07	0.16
*Magnolipollis*	0.66	/	1.48
*Proteacidites*	0.44	/	0.98
*Brucea*	0.04	/	0.08
*Symplocospollenites*	0.04	/	0.08
**Cold-tolerant elements**	37.43	16.7	62.87
*Pinuspollenites*	35.48	14.83	60.82
*Abiespollenites*	0.99	1.54	0.33
*Piceapollis*	0.96	0.33	1.72
**Xerophilous elements**	4.68	3.68	5.9
*Ephedripites*	3.83	2.74	5.16
*Artemisiaepollenites*	0.37	0.67	/
*Chenopodipollis*	0.48	0.27	0.74
**Hygrophilous elements**	4.3	3.33	5.49
*Alnipollenites*	3.68	2.2	5.49
*Taxodiaceaepollenites*	0.44	0.8	/
*Potamogetonacidites*	0.07	0.13	/
Pediastraceae	0.07	0.13	/
*Cyperaceaepollis*	0.04	0.07	/

/, RA less than 0.04%.

**Figure 4:**
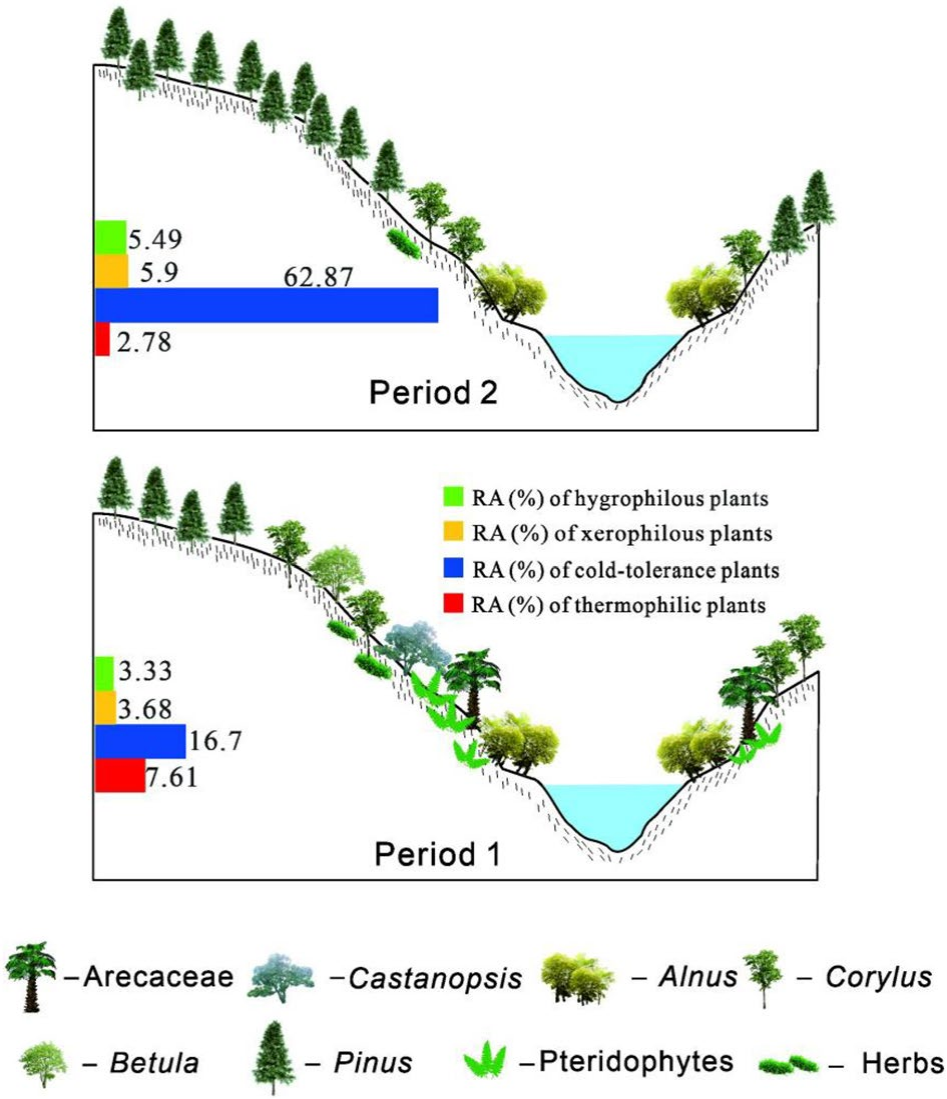
Reconstruction of the vegetation based on the pollen diagram of the Luanchuan section from the Late Palaeocene to the Early Eocene and the relative abundance (RA) (%) of each plant (red: thermophilic plants; blue: cold-tolerant plants; yellow: xerophilous plants; green: hygrophilous plants) with classification by plant biological habits in two zones.

**Figure 5:**
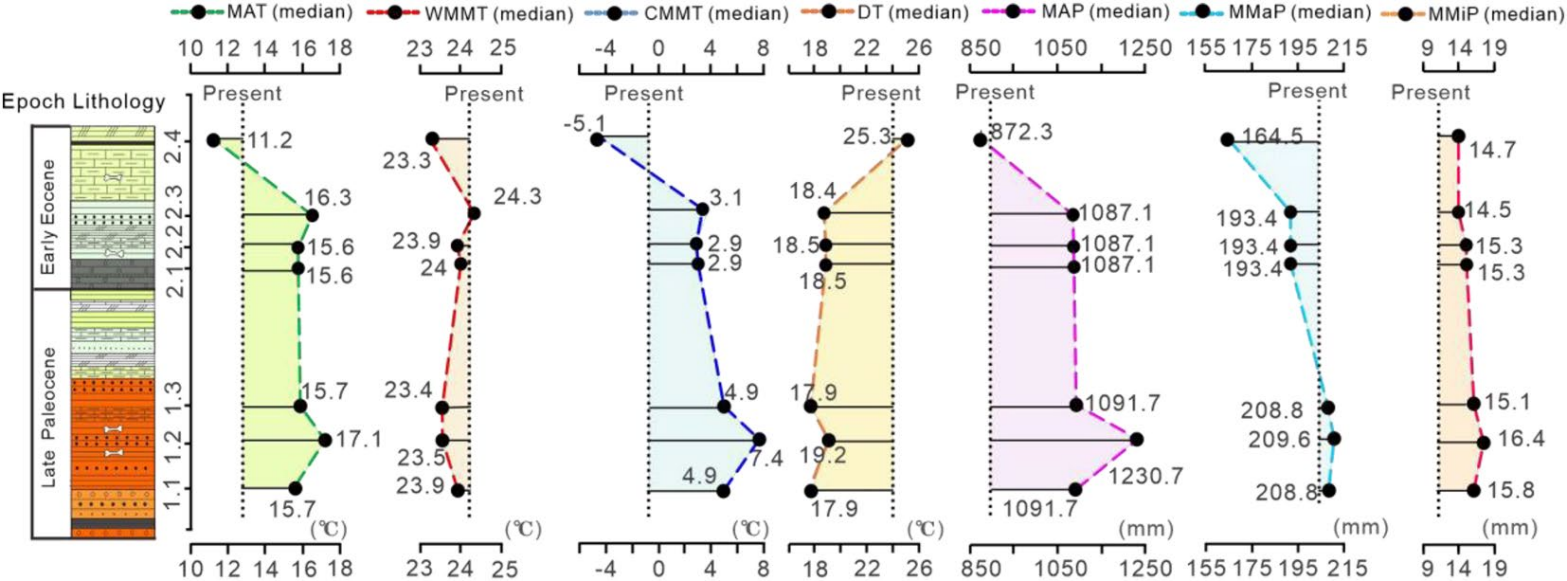
Comparison of the palaeoclimates of the seven periods (Periods 1.1–2.4) from the Late Palaeocene to Early Eocene and the current climate with median values (1.1–2.4: Periods 1.1–2.4).

In zone 1 (Sample Numbers LC1-LC23), we observed 1497 pollen grains and spores that belonged to 40 palynomorphs: 22 trees and shrubs (42.48%), 5 herbs (3.95%), 6 pteridophytes (30.26%), 5 ambiguous elements (23.05%) and 2 aquatic plants (0.26%) (Table 1; Figs. 4 and 5). Trees and shrubs were the dominant vegetation types in this zone. There were 10 thermophilic elements, three cold-tolerant elements, three xerophilous elements and five hygrophilous elements (Table 1). In total, the RA of the thermophilic elements was 7.61%, while the RAs of the cold-tolerant elements, xerophilous elements and hygrophilous elements were 16.70%, 5.90% and 5.49%, respectively.

In zone 2 (sample number LC24-LC44), we observed 1220 pollen grains and spores that belonged to 26 palynomorphs: 16 trees and shrubs (85.41%), 5 herbs (10.16%), 3 pteridophytes (2.05%) and 2 ambiguous elements (2.38%). Trees and shrubs dominated in this zone, and both pteridophytes and ambiguous elements were present in significantly lower numbers than in other zones. Aquatic plants disappeared in this zone (Table 1; Figs. 4 and 5). The thermophilic elements in this zone decreased to 2.87%, while the cold-tolerant elements increased to 62.87%. Xerophilous elements and hygrophilous elements increased to 5.90% and 5.49%, respectively (Table 1 and Figs. 4 and 5).

### Palaeovegetation

The whole palynological assemblage suggests that the palaeovegetation in LC during the Late Palaeocene–Early Eocene was a mixed forest of coniferous and deciduous broad-leaved trees (primary contents: *Betula*, *Corylus* and *Alnus*) under warm temperate conditions. Deciduous broad-leaved trees accounted for a certain proportion in the Late Palaeocene but decreased in the Early Eocene in LC (Figs. 4 and 5). In summary, among the four types of deciduous broad-leaved forest vegetation in China^35^, the vegetation types from the Late Palaeocene to Early Eocene in LC are the most similar to warm temperate mixed forests.

According to the palynological data from the two zones and the ecological distribution characteristics of each taxon, the vegetation succession in LC during the Late Palaeocene to Early Eocene is divided into two periods and described as follows (Fig. 4).

In Period 1 (zone 1, Late Palaeocene), 13 kinds of tropical and subtropical evergreen broad-leaved trees (primary contents: Arecaceae) appeared, with luxuriant fern growth (mainly Hemionitidaceae and Dennstaedtiaceae) in the understorey. Deciduous broad-leaved trees dominated in the mixed needle- and broad-leaved forest during this period, and the vegetation was characterized by rich plant diversity. Thermophilic elements (Cupressaceae, *Castanopsis*, Sapindaceae, Arecaceae, Myrtaceae, *Huodendron*, Araliaceae, Hamamelidaceae, and Rutaceae) and cold-tolerant elements (*Pinus, Abies* and *Picea*) developed in this period. Hygrophilous elements grew sparsely around a palaeolake or surrounding wetland^36^. Pediastraceae typically grew in lakes or swamps, and Potamogetonaceae sank or floated in freshwater. Xerophilous elements (Amaranthaceae, *Artemisia* and *Ephedra*) may have occurred on dry slopes (Fig. 4). In general, the climate during this stage was warm and moist.

In Period 2 (zone 2, Early Eocene), compared with Period 1, the plant diversity decreased, and only 26 palynomorph taxa occurred. The conifers replaced the deciduous broad-leaved trees to become the dominant group in the mixed coniferous and deciduous broad-leaved mixed forest in this period (Fig. 4). Thermophilic elements (from 7.61 to 2.78%) decreased by 4.83%. A few typical thermophilic elements (Cupressaceae, Arecaceae, Sapindaceae, Myrtaceae, Hamamelidaceae, Araliaceae, *Huodendron* and Rutaceae) disappeared, but Magnoliaceae, *Brucea,* Proteaceae and Symplocaceae emerged (Table 1). Cold-tolerant elements (from 16.70 to 62.87%) increased by 46.17% to become the main floral type in this period. At the same time, xerophilous elements (from 3.68 to 5.90%) increased by 2.22%. Only one hygrophilous element (*Alnus*) appeared in Period 2, with RAs increasing by 2.16% (from 3.33 to 5.49%).

### Palaeoclimate: quantitative climate reconstruction

Based on the NLRs of 37 seed plant taxa from the whole palynomorph assemblage (Table 2), seven climatic parameters of the LC section were obtained through the CA (Fig. S1): MAT values from 11.8 to 19.6 °C; WMMT values from 19.8 to 28 °C; CMMT values from 3.9 to 5.9 °C; DT values from 12.3 to 23.4 °C; MAP values from 793.9 to 1389.4 mm; MMaP values from 172.4 to 245.2 mm; and MMiP values from 8.0 to 22.1 mm.

**Table 2:** List of palynomorphs from the Late Palaeocene to Early Eocene in the Tantou Basin and their nearest living relatives (NLRs) (Wu and Ding^37^; Zhang et al.^36^).

Palynomorph taxa	Dazhang Fm	Tantou Fm	NLR	
			Previous name	Current name
*Pinuspollenites*	+	+	*Pinus*	*Pinus*
*Abiespollenites*	+	+	*Abies*	*Abies*
*Piceapollis*	+	+	*Picea*	*Picea*
*Taxodiaceaepollenites*	+		Taxodiaceae	Cupressaceae
*Ephedripites*	+	+	*Ephedra*	*Ephedra*
*Castanopsis*	+		*Castanopsis*	*Castanopsis*
*Alnipollenites*	+	+	*Alnus*	*Alnus*
*Ulmipollenites*	+	+	*Ulmus*	*Ulmus*
*Momipites coryloides*	+	+	*Corylus*	*Corylus*
*Juglanspollenites*	+	+	*Juglans*	*Juglans*
*Quercoidites*	+	+	*Quercus*	*Quercus*
*Symplocospollenites*		+	*Symplocos*	*Symplocos*
*Brucea*		+	*Brucea*	*Brucea*
*Huodendron*	+		*Huodendron*	*Huodendron*
*Caryapollenites*	+	+	*Carya*	*Carya*
*Pterocaryapollenites*	+		*Pterocarya*	*Pterocarya*
*Lonicerapollis*	+		Caprifoliaceae	Caprifoliaceae
*Sapindaceidites*	+		Sapindaceae	Sapindaceae
*Myrtaceidites*	+		Myrtaceae	Myrtaceae
*Oleoidearumpollenites*		+	Oleaceae	Oleaceae
*Proteacidites*	+	+	Proteaceae	Proteaceae
*Araliacoipollenites*	+		Araliaceae	Araliaceae
*Hamamelidacidites*	+		Hamamelidaceae	Hamamelidaceae
*Magnolipollis*		+	Magnoliaceae	Magnoliaceae
*Cyperaceaepollis*	+		Cyperaceae	Cyperaceae
*Graminidites*		+	Gramineae	Poaceae
*Umbelliferaepites*		+	Umbelliferae	Apiaceae
*Chenopodipollis*	+	+	Chenopodiaceae	Amaranthaceae
*Leguminidites*	+		Leguminosae	Fabaceae
*Monocolpopollenites*	+		Palmae	Arecaceae
*Palmaepollenites*	+		Palmae	Arecaceae
Urticaceae		+	Urticaceae	Urticaceae
*Potamogetonacidites*	+		Potamogetonaceae	Potamogetonaceae
*Rutaceoipollis*	+		Rutaceae	Rutaceae
*Corsinipollenites*	+		Onagraceae	Onagraceae
*Betulaceoipollenites*	+	+	*Betula*	*Betula*
*Cupuliferoipollenites*	+	+	*Castanea*	*Castanea*
*Artemisiaepollenites*	+		*Artemisia*	*Artemisia*
*Integricorpus*	+		*Integricorpus*	*Integricorpus*
*Tricolporopollenites*	+	+	*Tricolporopollenites*	*Tricolporopollenites*
*Pterisisporites*	+		*Pteris*	*Pteris*
Athyrium	+		Athyrium	Athyrium
Dennstaedtiaceae	+	+	Dennstaedtiaceae	Dennstaedtiaceae
Selaginellaceae	+		Selaginellaceae	Selaginellaceae
Hemionitidaceae	+	+	Hemionitidaceae	Hemionitidaceae
*Leiotriletes*	+	+	*Leiotriletes*	*Leiotriletes*
Pediastraceae	+		Pediastraceae	Pediastraceae
*Psophosphaera*	+	+	*Psophosphaera*	*Psophosphaera*

*Fm*. formation; *NLR* nearest living relative.

Based on Periods 1 and 2 (Table S2), we subdivided the palaeoclimatic changes into 7 periods, namely, Period 1.1, Period 1.2, Period 1.3, Period 2.1, Period 2.2, Period 2.3 and Period 2.4 (Table 3 and Table S3). Then, we quantitatively reconstructed curves showing the palaeoclimate changes from the Late Palaeocene to the Early Eocene (Fig. 5).

**Table 3:** Comparison of climate parameters from the Late Palaeocene to Early Eocene in Luanchuan with those of the present^22^ based on the median value of each climate parameter.

Climate parameters	The whole section	The Late Palaeocene	The Early Eocene	Subperiod							Present
		(Period 1)	(Period 2)	1.1	1.2	1.3	2.1	2.2	2.3	2.4	
MAT (°C)	15.7	15.7	15.6	15.7	17.1	15.7	15.6	15.6	16.3	11.2	12.2
WMMT (°C)	23.9	23.9	23.9	23.9	23.5	23.4	24	23.9	24.3	23.3	24.2
CMMT (°C)	4.9	4.9	3.1	4.9	7.4	4.9	2.9	2.9	3.1	− 5.1	− 0.8
DT (°C)	17.9	17.9	18.5	17.9	19.2	17.9	18.5	18.5	18.4	25.3	25
MAP (mm)	1091.7	1091.7	1087.1	1091.7	1230.7	1091.7	1087.1	1087.1	1087.1	872.3	880
MMaP (mm)	208.8	208.8	193.4	208.8	209.6	208.8	193.4	193.4	193.4	164.5	205.5
MMiP (mm)	15.1	15.1	14.5	15.8	16.4	15.1	15.3	15.3	14.5	14.7	9.9

## Discussion

At present, exposures of continuous Late Palaeocene to Early Eocene strata in China are very limited, and few studies have quantitatively analysed the palaeoclimate of this period with palynological methods. Our palynological assemblages are comparable to records from the Wutu Formation (42.50°N, 127.17°E) (Early Eocene) in Shandong Province, East China^36^. Overall, both sites are characterized by high percentages of conifer palynomorph taxa, such as *Pinuspollenites* (*Pinus*)*, Abiespollenites* (*Abies*) and *Piceapollis* (*Picea*), accompanied by relatively frequent broad-leaved tree pollen represented by *Ulmipollenites* (*Ulmus*), *Quercoidites* (*Quercus*) and *Betulaceoipollenites* (*Betula*), as well as low percentages of fern spores. This similarity in taxonomic composition suggests that the two palynofloras may be close in age.

In reviewing a large number of studies, we also find that the pollen assemblages in this study are strongly comparable to those of the strata in the Lizigou Formation to the Guchengzi Formation (Late Palaeocene–Early Eocene) in the Fushun Basin^19,38^. Overall, the vegetation at both sites was characterized by coniferous broad-leaved mixed forests, indicating a warm and humid climate. Both sites feature the pollen of thermophilic taxa, such as *Castanopsis*, *Palmaepollenites* (Arecaceae), *Pterocaryapollenites* (*Pterocarya*) and *Rutaceoipollenites* (Rutaceae); broad-leaved tree taxa represented by *Ulmipollenites* (*Ulmus*), *Quercoidites* (*Quercus*) and *Juglanspollenites* (*Juglans*); conifers, such as *Piceapollenites* (*Picea*) and *Pinuspollenites* (*Pinus*); and aquatic taxa, such as *Taxodiaceaepollenites* (Cupressaceae) and *Alnipollenites* (*Alnus*). However, the pollen of thermophilic taxa appears only in the Dazhang Formation (Late Palaeocene), which differs from the situation in the Fushun Basin. The vegetation types reflected in other palynological records are also strongly comparable to the mixed conifer and broad-leaved forests in this study. The above analyses further confirm the Late Palaeocene and the Early Eocene sedimentary ages of Dazhang Fm. and Tantou Fm. in the Tantou Basin.

The palaeoclimate change throughout the whole section went through seven stages (Fig. 5). The first three stages occurred in the Late Palaeocene. The MAT (median values from 15.7 to 17.1 to 15.7 °C) and CMMT (from 4.9 to 7.4 to 4.9 °C) first increased and then decreased (Table 3; Fig. 5). These results suggest that there was a short period of extreme warming during the Late Palaeocene. The other four stages occurred in the Early Eocene. The MAT (from 15.6 to 15.6 to 16.3 to 11.2 °C) and CMMT (from 2.9 to 2.9 to 3.1 to − 5.1 °C) generally exhibited an initial increase and then a decrease (Table 3; Fig. 5). Period 2.3 was the hottest time during the Early Eocene, which may indicate the occurrence of the Early Eocene Climatic Optimum (EECO) (Fig. 5). However, the palaeotemperature curves of the whole section showed a trend of cooling, with the annual and winter temperatures dropping by 4.5 °C and 10 °C from bottom to top, respectively (Fig. 5). In particular, the sudden decreases in MAT and CMMT from Period 2.3 to Period 2.4 may imply global cooling during the Early Eocene. However, the DT values increased by 7.4 °C from bottom to top (Fig. 5), which indicate that the seasonal differences increased. Overall, the Late Palaeocene was hotter than the Early Eocene, while the seasonal differences strengthened from the Late Palaeocene to the Early Eocene (Table 3; Table S2; Fig. 5).

The comparison between the palaeotemperature and modern data from the LC (Table 3, Fig. 5) indicates that compared to the other periods, the last period was much more similar to the current climate conditions, which are dominated by a monsoon climate (Table 3, Fig. 5). The MAT (11.2 °C) in the last period was lower than that at present (12.2 °C) by 1 °C; the WMMT (23.3 °C) was lower than that at present (24.2 °C) by 0.9 °C; the CMMT (− 5.1 °C) was lower than that at present (− 0.8 °C) by 4.3 °C; and the DT (25.3 °C) was higher than that at present (25 °C) by 0.3 °C. These phenomena most likely imply the emergence of a monsoonal climate or monsoon-like climate during the Early Eocene. The results of Shukla et al.^3^, who used a multivariate foliar physiognomic analysis to study several tens of thousands of years of deposition in the Gurha Mine (27.87°N, 72.87°E) in Rajasthan, India, also indicate a pronounced monsoon climate signature during the Early Eocene. Our results are consistent with theirs. Our results are also consistent with those of Caves Rugenstein and Page Chamberlain^4^, who used carbon isotope analysis to reconstruct climate evolution in Asia during the Cenozoic and indicated that atmospheric circulation has been similar to that today for at least 55 million years.

The MAP increased by 139 mm (median values: from 1091.7 to 1230.7 mm) from Period 1.1 to 1.2 (Fig. 5). Then, the MAP decreased (from 1230.7 to 872.3 mm) (Fig. 5), and the most obvious drop in the MAP (from 1087.1 to 872.3 mm) occurred in the last stage (from Period 2.3 to Period 2.4) (Fig. 5). Additionally, from Period 1.1 to Period 1.3, the MMaP remained stable (Fig. 5) and then dropped by ~ 15 mm (from 208.8 to 193.4 mm) from Period 1.3 to Period 2.3, which reflected a drying trend (Fig. 5). A remarkable decrease in MMaP (~ 30.0 mm) from Period 2.3 to Period 2.4 reflected a drying trend. The MMiP of the whole section showed no major fluctuation (Fig. 5).

In conclusion, the precipitation data reveal a local aridification trend, especially in the Early Eocene (Fig. 5). However, the MAP (> 800 mm) of the whole section reflects a humid climate, although it experienced a drying trend. The remarkable decrease in temperature and precipitation that occurred during the Early Eocene might be related to global cooling and/or the regional geological structure, but the climate condition changed to become very similar to that of today with a monsoon climate, which might imply or be related to the emergence/strengthening of the East Asian monsoon or monsoon-like climate. This humid climate (MAP > 800 mm) in the Early Eocene continued to the Early-Middle Eocene in this basin^16^. Our results are different from previous palaeoenvironmental reconstructions depending on the sedimentary rock characteristics, which concluded that Asia was dominated by a zonal climate pattern in the Early Eocene epoch, with an almost latitudinal arid/semiarid band throughout central China (20–40°N palaeolatitude)^33^. This long-standing model has recently been challenged by growing multidisciplinary evidence. Some previous studies indicated that middle China definitely fluctuated between humid and relatively dry climates during the Early Eocene, as seen in the modern regime of the Eastern Asian monsoon^33^. Therefore, the origin of the East Asian monsoon can be traced back to the Eocene, especially the Early Eocene, pushing the proposed age back by approximately 22 million years^39^. In addition, based on multidisciplinary evidence, Quan et al.^2^ analysed the climate of China during the Early Eocene and indicated that the data seem to reflect a humid climate in middle China during the Early Eocene. Therefore, they suggested that a monsoonal or monsoon-like climate might have prevailed over China during the Early Eocene.

The global environment has changed significantly since the start of the Cenozoic^40,41^. At the same time, a series of tectonic movements has also had a significant impact on the global environment. For example, the uplift of the Tibetan Plateau is considered to be an important factor in the formation of the Asian monsoon in the Cenozoic^6,42,43^. However, the emergence/origin of the Asian monsoon remains poorly constrained due to a lack of well-dated records^6^. Based on carbon isotope analysis, Caves Rugenstein and Page Chamberlain^4^ reconstructed the Cenozoic climate evolution in Asia and indicated that atmospheric circulation has remained similar to that of today for at least 55 million years. However, some research has pointed out that the climate pattern in China is mainly controlled by the planetary wind system, and a broad east–west arid zone formed between 18 and 35°N during the Late Palaeocene to Early Eocene^1,8,44^, while the humid region in southern East Asia, now dominated by monsoons, was also drier at that time^9^. By using palaeobotanical methods, some research has investigated the origin of the Asian monsoon and indicated that the Asian monsoon might have existed in the Early Eocene^3,38,39^. Recently, Licht et al.^7^ proposed that the East Asian monsoon most likely existed in the late Eocene. However, Li et al.^8^ used the low-resolution Norwegian Earth System Model (NorESM-L) and the high-resolution Community Atmosphere Model version 4 (CAM4) to simulate the climate in China during the Eocene and evaluated the climatic effects of topography and sea surface temperature (SST) on the East Asian climate. They seem to support the presence of a zonal/zonal-like arid desert/steppe climate band in central East Asia in the Early Eocene.

In general, whether the Early Eocene climate model was controlled by a zonal climate pattern or by a nonzonal pattern is still unknown. The lack of an extensive arid zone in central China during the Early Eocene is usually the main factor for judging the existence of a monsoonal climate or monsoon-like climate. Our results indicated that central China experienced an obvious decrease in precipitation from the Late Palaeocene to the Early Eocene. However, the whole section was humid with an MAP no less than 800 mm which was also supported by the climatic characteristics of lithologic sedimentary reactions in this region (Fig. 2), representing a different conclusion than previous palaeoenvironmental reconstruction studies dependent on sedimentary rock characteristics. In addition, previous work indicated that the Paleogene climate in East Asia was controlled by a zonal climate pattern (planetary wind system) that caused the climate of central China to be arid, with an MAP less than 500 mm based on the massive deposition of red beds and evaporites in the sediments at that time^2^. However, red beds and evaporites are not necessarily indicative of an arid environment^2^. Therefore, the conclusion that the climate of the Palaeogene was controlled by the planetary wind system seems unreliable, especially during the Early Eocene. Our results indicated a warm and humid climate throughout the Late Palaeocene to the Early Eocene (MAP > 800 mm), and a sudden climate change occurred in the Early Eocene, with the MAT and MAP decreasing by 5.1 °C and 214.8 mm, respectively, and becoming very similar to those of the present climate, which is controlled by the monsoon. Thus, this change may signal the emergence of the EAM in East Asia (Fig. 5). In addition, this warm and humid climate in the Early Eocene in the Tantou Basin, central China, continued until the Early-Middle Eocene in this basin^22^. Therefore, this study supports the hypothesis that a monsoon or monsoon-like climate may have emerged/developed in the early Palaeogene in East Asia. The emergence/origin of the EAM in East Asia may have occurred in the Early Eocene. However, considering the complex factors of monsoon climate formation, it is difficult for current research methods to accurately simulate past regional structures and landscapes. Therefore, to better understand past climate changes, a large number of multidisciplinary studies are still needed.

## Conclusions

1. The vegetation in the Tantou Basin experienced two major successional stages from the Late Palaeocene to the Early Eocene. The first stage was a coniferous and deciduous broad-leaved mixed forest with a warm and humid climate dominated by deciduous broad-leaved trees. The second stage was the same mixed forest but dominated by conifers, with a relatively cool and dry climate.
2. The palaeoclimates of the Late Palaeocene and Early Eocene in the Tantou Basin were quantitatively reconstructed by using the CA method. During the Late Palaeocene, the MAT values were 11.8–19.6 °C, the WMMT values were 19.8–28 °C, the CMMT values were 3.9–5.9 °C, the DT values were 12.3–23.4 °C, the MAP values were 793.9–1389.4 mm, the MMaP values were 172.4–245.2 mm, and the MMiP values were 8–22.1 mm. During the Early Eocene, the MAT values were 8.5–22.7 °C, the WMMT values were 19.8–28.0 °C, the CMMT values were 0.2–5.9 °C, the DT values were 12.3–24.6 °C, the MAP values were 784.7–1389.4 mm, the MMaP values were 141.5–245.2 mm, and the MMiP values were 6.9–22.1 mm.
3. The early Paleogene climate in East Asia dominated whether by zonal climate or non-zonal monsoon climate patterns has persisted for decades. In this study, seven samples that contain more than 100 pollen grains with more than 10 pollen taxa indicated a wetter climate in the Tantou Basin, central China, during the Late Palaeocene to Early Eocene. These results further refuted East Asia climate in this period was controlled by a zonal climate pattern (planetary wind system) to cause the arid climate in central China, but suggesting the possible existence of a monsoon or monsoon-like climate during this period. Additionally, we found that the climate became increasingly similar to the current monsoon-controlled climate by comparing seven palaeoclimatic records with the present climate. In particular, the palaeoclimate during the last period in the Early Eocene was the most similar to today’s, which probably indicates the emergence of the monsoon during the Early Eocene.

## Materials and methods

### Materials

This study focused on 44 palynological sample blocks from the upper Palaeocene to the lower Eocene series, including the Gaoyugou Fm., Dazhang Fm., and Tantou Fm., in the Tantou Basin at an elevation of 500 m (34° 01′1.2ʺ N, 111° 46′ 1.2ʺ E) (Fig. 1). The sporopollen sampling site is shown in detail in Fig. 2. Sample numbers LC1 to LC3 (LC1–3) were collected from the Gaoyugou Fm., yielding zero spores and pollen grains; LC4–23 were collected from the Dazhang Fm., yielding 1497 spores and pollen grains; and LC24–44 were collected from the Tantou Fm., yielding 1220 spores and pollen grains (Raw data in Supplementary material).

### Methods

The palynological samples were analysed by the heavy liquid separation method (density = 2.1 g/ml)^45,46^ and observed under a Leica DM 2500 microscope. The main palynomorphs were photographed under a Leica DM 2500 microscope, and 48 palynomorphs were identified. The single-grain technique^47^ was applied to obtain high-quality images using an FEI Quanta 200 environmental scanning electron microscope (Figs. S1-S6). To calculate the palynomorph relative abundance (RA) of a taxon, the equation RA = N/Nt was used, where N is the pollen/spore number of a taxon and Nt is the total pollen/spore number of all taxa combined in the pool of samples^48^. The palynomorph taxa were grouped according to the ecological requirements of their nearest living relatives (NLRs)^49^. TILIA and TILIAGRAPH software were used to construct the pollen diagram (Fig. 3).

In this study, we used the CA^50^ to quantitatively reconstruct the palaeoclimate in LC. The CA is widely used, and it was the first method tested with modern vegetation^50^. This method has large uncertainties^51^ because most of the miospore components in the Mesozoic and Palaeogene palynofloras are extinct, making it difficult or impossible to correlate them with modern genera or even families^8^. However, a series of studies have been carried out by applying the CA^13,52,53^, with the Palaeoflora Database being updated constantly^54^.

By employing the CA, we first searched and determined the NLRs of seed plants in the palynological assemblage (Table 2) and then determined the geographic distributions of all NLRs^51^. This method allowed us to obtain the coexistence intervals of seed plants in the palynological assemblage and climatic parameters. Here, we established seven climatic parameters: the mean annual temperature (MAT), mean temperature of the warmest month (WMMT), mean temperature of the coldest month (CMMT), difference in temperature between the coldest and warmest months (DT), mean annual precipitation (MAP), maximum monthly precipitation (MMaP) and mean minimum monthly precipitation (MMiP). Climate data were extracted from the Information Department of the Beijing Meteorological Center and partially from European data^54^. In addition, we used only samples that contained more than 100 grains for pollen analyses^55^. Among them, we collected samples with more than 11 seed plant taxa^50^ to quantitatively reconstruct the palaeoclimate more accurately. We then constructed curves of temperature and precipitation changes for the whole section.

## Supplementary Information

Supplementary Information.

## Data Availability

The datasets used and/or analysed during the current study are available from the corresponding author on reasonable request.
