# Supplementary material for: New evidence of the emergence of the East Asian monsoon in the early Palaeogene
Source: Sci Rep. 2022 Nov 28;12:20471. doi: 10.1038/s41598-022-24298-z (PMC9705385; doi:10.1038/s41598-022-24298-z)
Supplement: Supplementary file 1 — Supplementary Information. [file 41598_2022_24298_MOESM1_ESM.pdf]

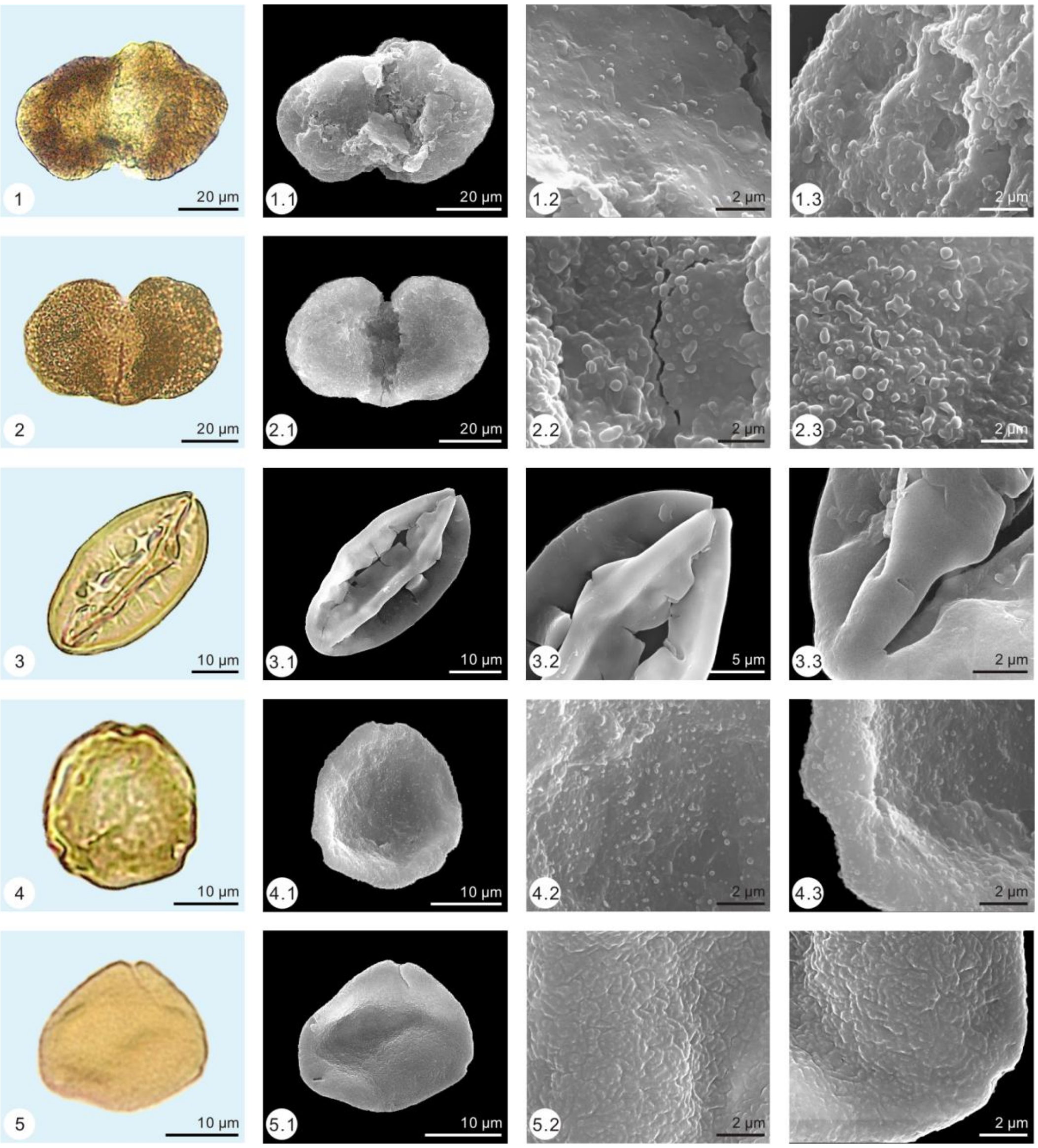

Fig S1. Pollen assemblage from the Late Paleocene to Early Eocene of Luanchuan. 1-2. *Pinuspollenites* (1.1. SEM picture of the previous grain; 1.2. show details of air sac; 1.3. show details of the body), 3. *Ephedripites*, 4-5. *Momipites Coryloides*.

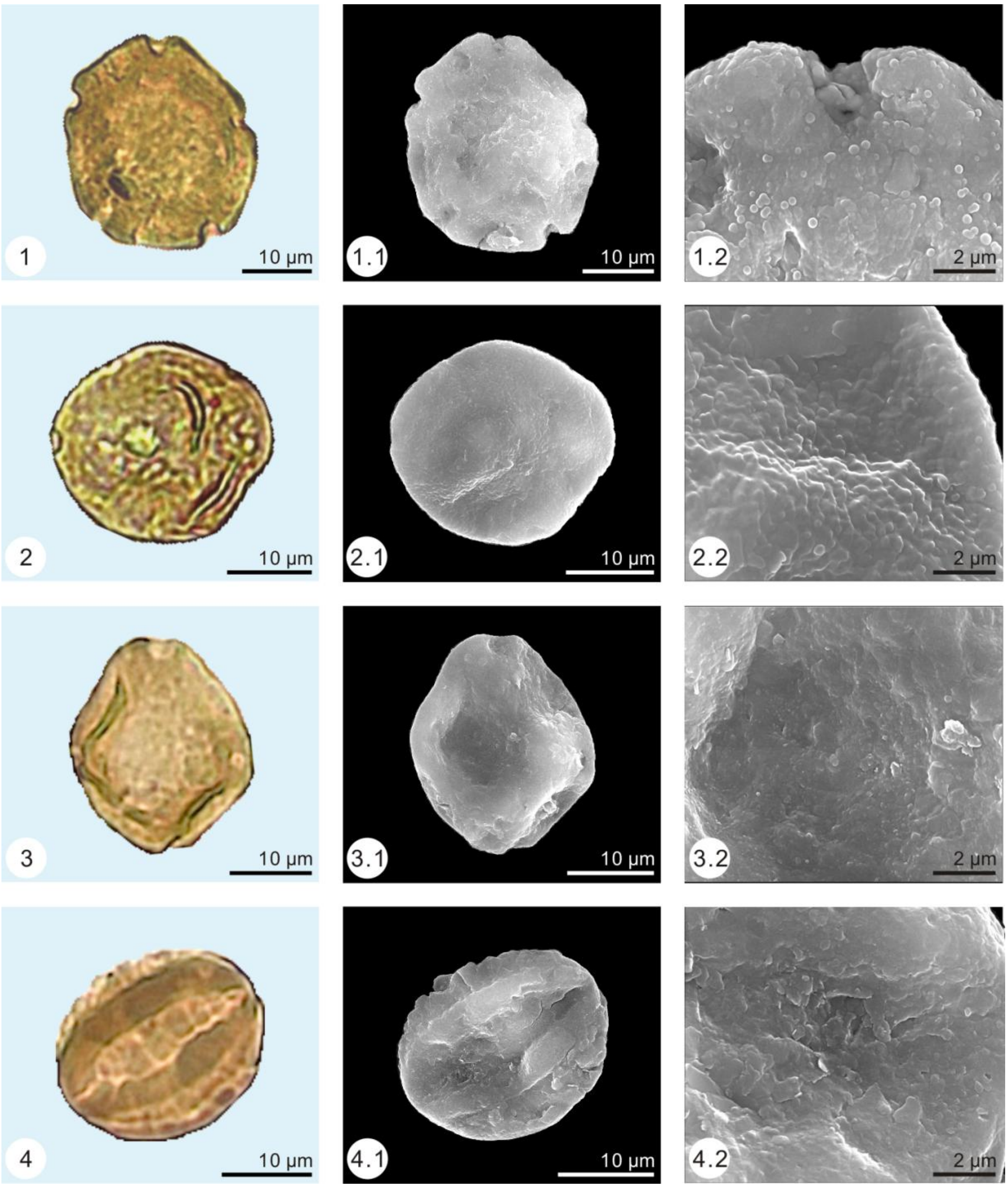

Fig S2. Pollen assemblage from the Late Paleocene to Early Eocene of Luanchuan. 1. *Pterocaryapollenites*, 2. *Caryapollenites*, 3. *Alnipollenites*, 4. *Castanopsis*.

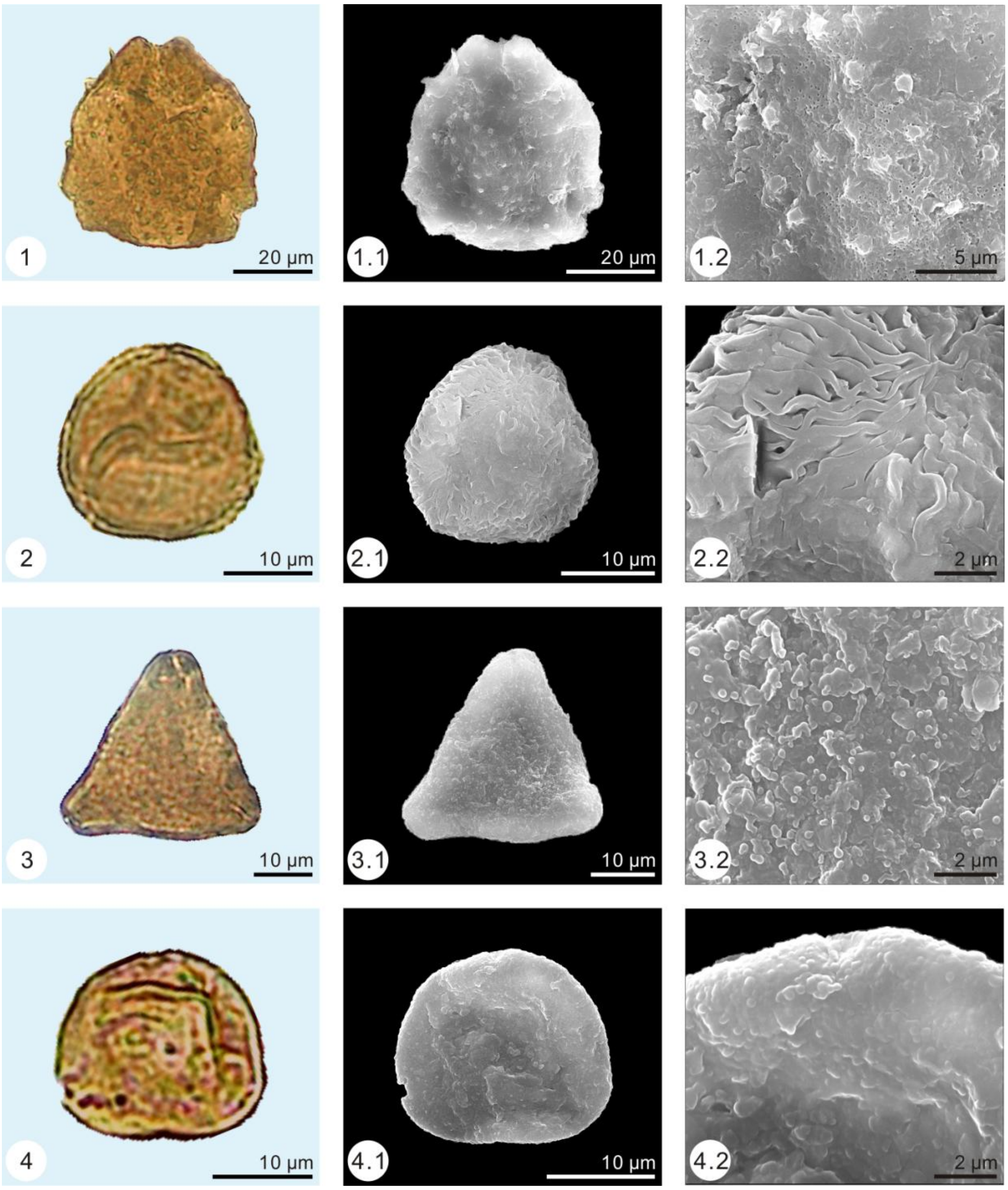

Fig S3. Pollen assemblage from the Late Paleocene to Early Eocene of Luanchuan. 1. *Lonicerapollis*, 2. *Brucea*, 3. *Sapindaceidites*, 4. *Urticaceae*.

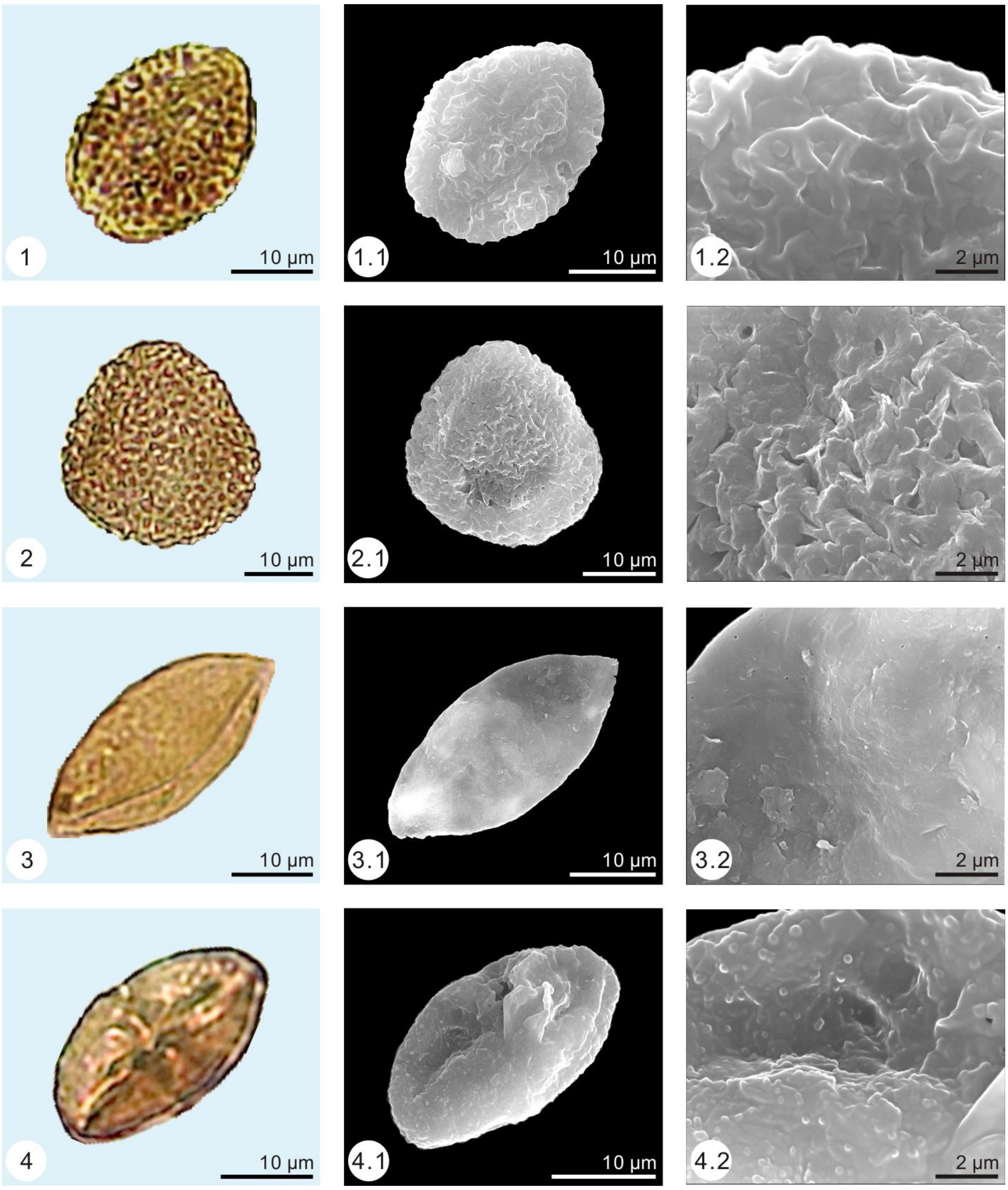

Fig S4. Pollen assemblage from the Late Paleocene to Early Eocene of Luanchuan. 1. *Oleoidearumpollenites*, 2. *Potamogetonacidites*, 3. *Magnolipollis*, 4. *Rutaceae*.

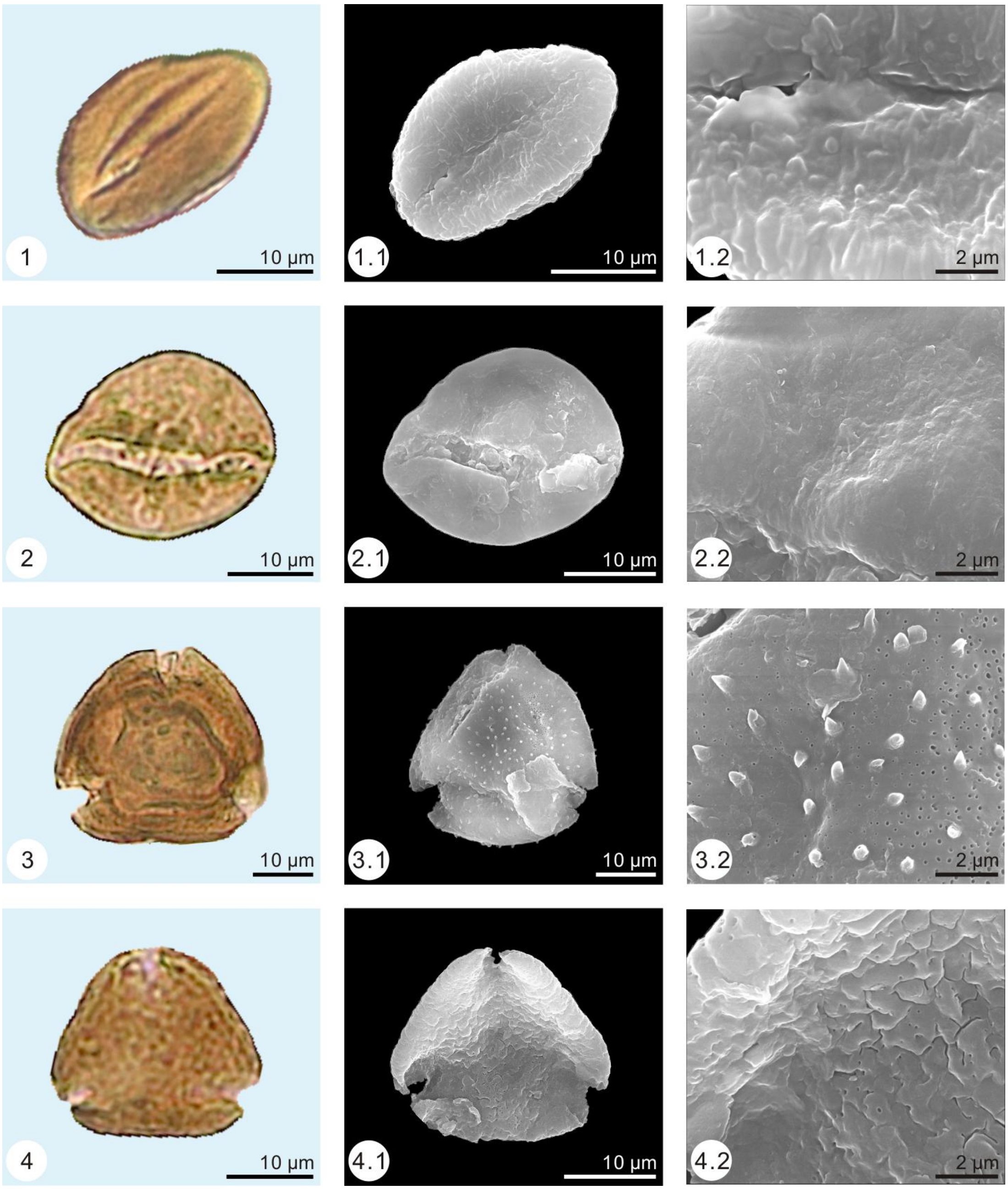

Fig S5. Pollen assemblage from the Late Paleocene to Early Eocene of Luanchuan. 1. *Monocolpopollenites*, 2. *Palmaepollenites*, 3. *Lonicerapollis*, 4. *Huodendron*.

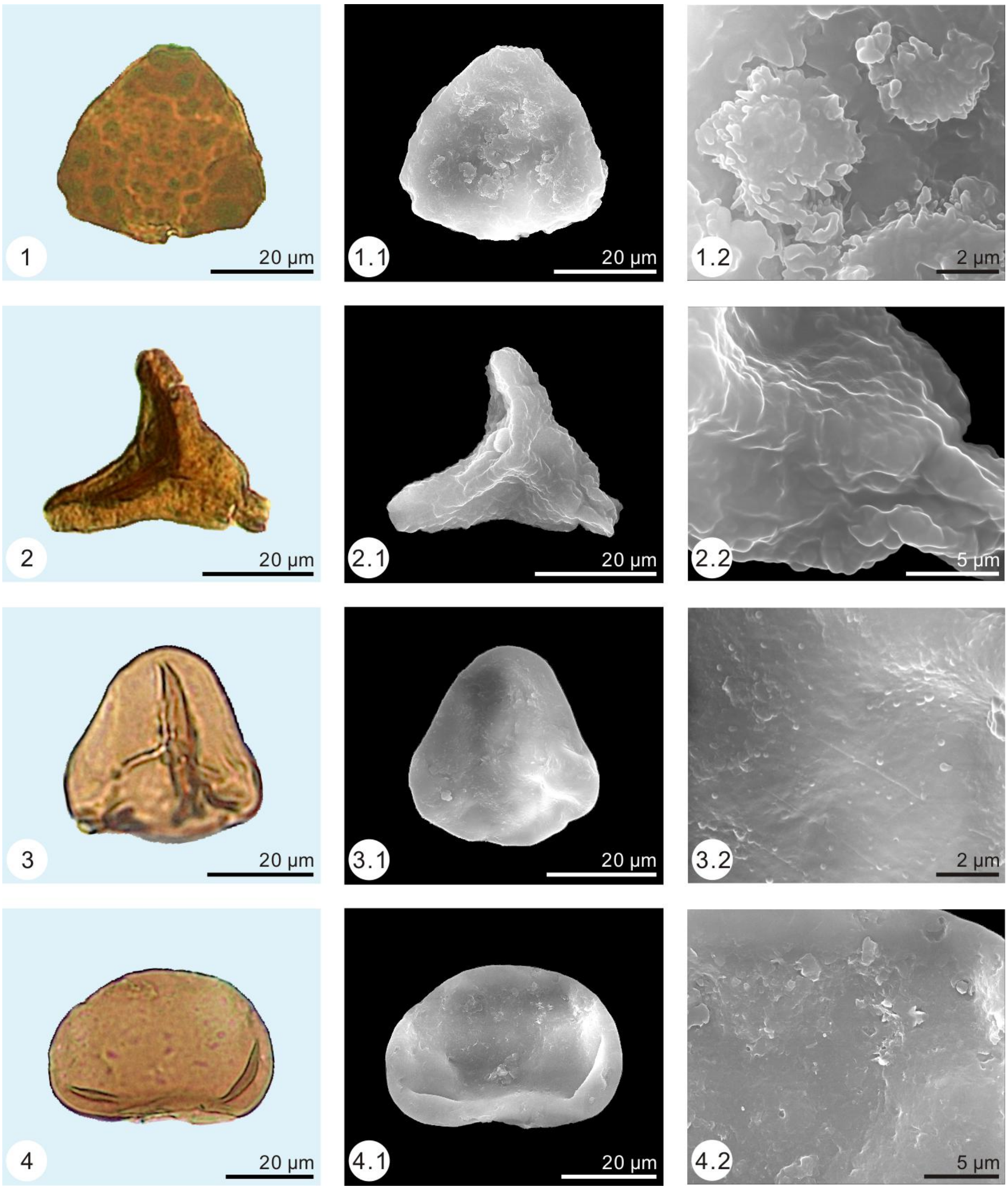

Fig S6. Pollen assemblage from the Late Paleocene to Early Eocene of Luanchuan. 1. *Corsinipollenites*, 2. *Integricorpus*, 3. Hemionitidaceae, 4. *Athyrium*.

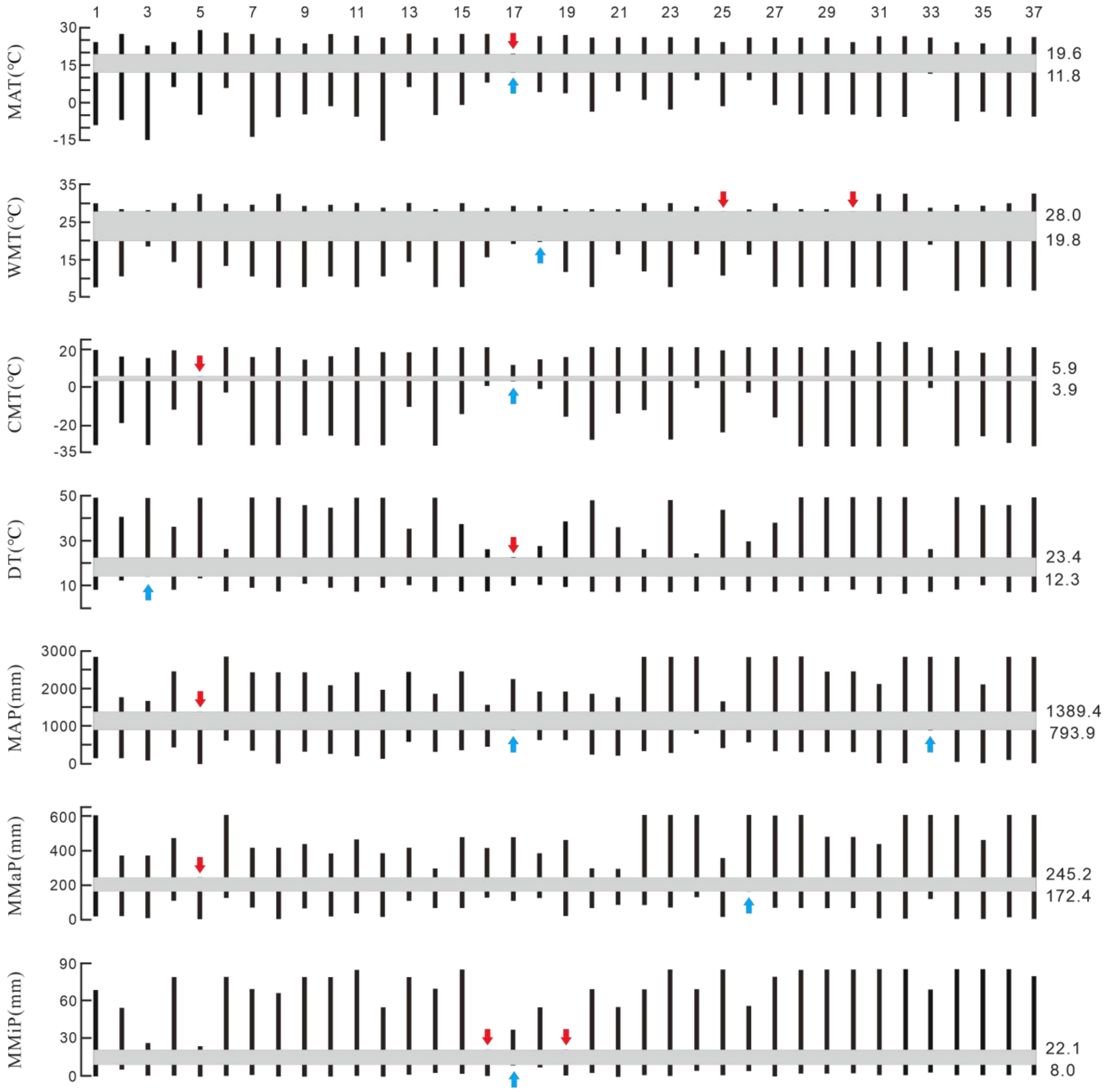

Fig. S7 Coexistence intervals of the climatic parameters of the nearest living relatives of the palynomorphs in Luanchuan section.

(1. *Pinus*; 2. *Abies*; 3. *Picea*; 4. Taxodiaceae; 5. *Ephedra*; 6. *Castanopsis;* 7. *Alnus*; 8. *Ulmus*; 9. *Corylus*; 10. *Juglans*; 11. *Quercus*; 12. *Betula;* 13.*Castanea*; 14. *Artemisia*; 15. *Symplocos*; 16. *Brucea*; 17.*Huodendron*; 18.*Carya*; 19.*Pterocarya;* 20. Caprifolaceae; 21. Sapindaceae; 22. Myrtaceae; 23. Oleaceae; 24. Proteaceae; 25. Araliaceae; 26. Hamamelidaceae; 27. Magnoliaceae; 28. Cyperaceae; 29.Gramineae; 30.Umbelliferae; 31.Chenopodiaceae; 32. Leguminosae; 33. Palmae; 34. Urticaceae; 35. Potamogetonaceae; 36. Rutaceae; 37. Onagraceae).

Table S1 List of the palynomorph RA of Luanchuan whole section and palynological zones from the Late Paleocene to Early Eocene.

Palynomorph	The whole section (%)	Palynological zones		Palynomorph	The whole section (%)	Palynological zones	
		Zone 1 (%)	Zone 2 (%)			Zone 1 (%)	Zone 2 (%)
**Trees and shrubs**	**61.76**	**42.48**	**85.41**	**Herbs**	**6.74**	**3.95**	**10.16**
Pinuspollenites	35.48	14.83	60.82	*Ephedripites*	3.83	2.74	5.16
Momipites coryloides	7.29	8.35	5.98	*Umbelliferaepites*	0.96	0	2.13
Ulmipollenites	3.9	5.74	1.64	*Graminidites*	0.88	0	1.97
Alnipollenites	3.68	2.2	5.49	*Chenopodipollis*	0.48	0.27	0.74
Betulaceoipollenites	1.88	2.67	0.9	*Artemisiaepollenites*	0.37	0.67	0
Quercoidites	1.58	0.94	2.38	*Corsinipollenites*	0.11	0.2	0
Juglanspollenites	1.47	0.53	2.62	Urticaceae	0.07	0	0.16
Abiespollenites	0.99	1.54	0.33	*Cyperaceaepollis*	0.04	0.07	0
Piceapollis	0.96	0.33	1.72	**Pteridophytes**	**17.6**	**30.26**	**2.05**
Castanopsis	0.7	1.27	0	Hemionitidaceae	6.55	10.75	1.39
Magnolipollis	0.66	0	1.48	Dennstaedtiaceae	5.19	8.95	0.57
Taxodiaceaepollenites	0.44	0.8	0	*Athyrium*	2.58	4.68	0
Proteacidites	0.44	0	0.98	*Leiotriletes*	1.77	3.14	0.08
Sapindaceidites	0.37	0.67	0	*Pterisisporites*	1.44	2.61	0

Palmaepollenites	0.37	0.67	0	*Selaginellaceae*	0.07	0.13	0
Myrtaceidites	0.33	0.6	0	**Other Elements**	**13.77**	**23.05**	**2.38**
Hamamelidacidites	0.29	0.53	0	*Tricolporopollenites*	10.71	17.57	2.3
Caryapollenites	0.29	0.07	0.57	*Monocolpopollenites*	2.17	3.94	0
Lonicerapollis	0.11	0.2	0	Leguminosae	0.59	1.07	0
Castanopsis	0.11	0.07	0.16	*Psophosphaera*	0.22	0.33	0.08
Huodendron	0.07	0.13	0	*Integricorpus*	0.07	0.13	0
Pterocaryapollenites	0.07	0.13	0	**Aquatic Plants**	**0.14**	**0.26**	**0**
Araliacoipollenites	0.07	0.13	0	Pediastraceae	0.07	0.13	0
Oleoidearumpollenites	0.07	0	0.16				
Brucea	0.04	0	0.08				
Symplocospollenites	0.04	0	0.08	*Potamogetonacidites*	0.07	0.13	0
Rutaceae	0.04	0.07	0				

/, RA less than 0.04%

Table S2 Comparison of climate parameters from the Late Paleocene to Early Eocene in Luanchuan.

Climate parameter	The whole section (The Late Paleocene to the Early Eocene)		Dazhang Formation (The Late Paleocene)		Tantou Formation (The Early Eocene)	
	Climate value (Median value)	Bordering taxa	Climate value (Median value)	Bordering taxa	Climate value (Median value)	Bordering taxa
MAT (°C)	11.8-19.6 (15.7)	*Huodendron*	11.8-19.6 (15.7)	*Huodendron*	8.5-22.7 (15.6)	*Brucea*/Proteaceae-*Picea*
WMMT(°C)	19.8-28.0 (23.9)	*Carya*-Araliaceae	19.8-28.0 (23.9)	*Carya*-Araliaceae	19.8-28.0 (23.9)	*Carya*-Umbelliferae
CMMT (°C)	3.9-5.9 (4.9)	*Huodendron-Ephedra*	3.9-5.9 (4.9)	*Huodendron-Ephedra*	0.2-5.9 (3.1)	*Brucea-Ephedra*
DT (°C)	13.3-23.4 (17.9)	*Picea-Huodendron*	13.3-23.4 (17.9)	*Picea-Huodendron*	12.3-24.6 (18.5)	*Picea*-Proteaceae
MAP (mm)	793.9-1389.4 (1091.7)	Palmae/*Huodendron-Ephedra*	793.9-1389.4 (1091.7)	Palmae/*Huodendron-Ephedra*	784.7-1389.4 (1087.1)	Proteaceae-*Ephedra*
MMaP (mm)	172.4-245.2 (208.8)	Hamamelidaceae-*Ephedra*	172.4-245.2 (208.8)	Hamamelidaceae-*Ephedra*	141.5-245.2 (193.4)	*Carya-Ephedra*
MMiP (mm)	8.0-22.1 (15.1)	*Carya-Pterocarya*	8.0-22.1 (15.1)	*Carya-Pterocarya*	6.9-22.1 (14.5)	*Carya-Brucea*

Table S3 The 7 Climate parameters of seven periods from the Late Paleocene to Early Eocene in Luanchuan.

Number of palynomorpy	Sample number	MAT(°C) (Median value)	WMMT(°C) (Median value)	CMMT(°C) (Median value)	DT(°C) (Median value)	MAP(mm) (Median value)	MMaP(mm) (Median value)	MMiP(mm) (Median value)
11	LC 40* (Period 2-4)	-0.4-22.7 (11.2)	18.4-28.2 (23.3)	-16-5.9 (-5.1)	12.3-38.2 (25.3)	355.2-1389.4 (872.3)	83.8-245.2 (164.5)	5.7-23.6 (14.7)
11	LC 34, 37* (Period 2-3)	8.5-24.0 (16.3)	19.8-28.8 (24.3)	0.2-5.9 (3.1)	12.1-24.6 (18.4)	784.7-1389.4 (1087.1)	141.5-245.2 (193.4)	6.9-22.1 (14.5)
13	LC 32* (Period 2-2)	8.5-22.7 (15.6)	19.8-28.0 (23.9)	-0.2-5.9 (2.9)	12.3-24.6 (18.5)	784.7-1389.4 (1087.1)	141.5-245.2 (193.4)	6.9-23.6 (15.3)
17	LC 30, 28* (Period 2-1)	8.5-22.7 (15.6)	19.8-28.2 (24.0)	-0.2-5.9 (2.9)	12.3-24.6 (18.5)	784.7-1389.4 (1087.1)	141.5-245.2 (193.4)	6.9-23.6 (15.3)
28	LC 11* (Period 1-3)	11.8-19.6 (15.7)	18.7-28.0 (23.4)	3.9-5.9 (4.9)	12.3-23.4 (17.9)	793.9-1389.4 (1091.7)	172.4-245.2 (208.8)	8.0-22.1 (15.1)
15	LC 8* (Period 1-2)	11.5-22.7 (17.1)	18.7-28.2 (23.5)	-0.2-14.9 (7.4)	12.3-26.0 (19.2)	793.9-1667.4 (1230.7)	137.3-281.9 (209.6)	5.7-27.0 (16.4)
20	LC 5*, 6 (Period 1-1)	11.8-19.6 (15.7)	19.8-28.0 (23.9)	3.9-5.9 (4.9)	12.3-23.4 (17.9)	793.9-1389.4 (1091.7)	172.4-245.2 (208.8)	8.0-23.6 (15.8)

Number*, with number of palynomorpy ≧11
